# Surgical clipping for a recurrent perforator aneurysm of the A1 segment following endovascular electrocoagulation therapy: a case report

**DOI:** 10.1186/s41016-026-00448-4

**Published:** 2026-09-10

**Authors:** Maogui Li, Zidong Wang, Qingke Cui, Donghai Wang

**Affiliations:** 1 Department of Neurosurgery, Qilu Hospital of Shandong University, Wenhuaxi Road, Jinan, 250012 China; 2 Shandong Key Laboratory of Brain Health and Function Remodeling, Jinan, 250012 China; 3 Department of Neurosurgery, Liaocheng People’s Hospital, Liaocheng, 252001 China

**Keywords:** Aneurysms, Electrocoagulation, Recurrence

## Abstract

**Background:**

Recently, endovascular electrocoagulation has been successfully used to treat ruptured aneurysms that cannot be catheterized with a microcatheter, but more research is needed to evaluate the efficacy of this technique. In this report, we present the first reported case of early aneurysm recurrence following electrocoagulation therapy and describe our experience of retreatment for this lesion.

**Case presentation:**

A 51-year-old female presented with spontaneous subarachnoid hemorrhage, who was diagnosed with a rare tiny aneurysm arising from the A1 segment of anterior cerebral artery. Because of the sharp turning angle of parent artery and the extremely narrow aneurysm neck, attempts at catheterization of the aneurysm sac were unsuccessful. Thus, pure micro-guidewire electrocoagulation was selected as the salvage treatment approach. Initially, the aneurysm was successfully occluded by electrocoagulation, but follow-up angiography at 5 weeks postoperatively showed recanalization of the lesion. Eventually, the recurrent aneurysm was safely obliterated by surgical clipping, providing a direct view of the pathological changes following electrocoagulation therapy.

**Conclusions:**

Endovascular electrocoagulation carries a risk of aneurysm recanalization despite initial success. Close angiographic follow-up is warranted to ensure the efficacy of this technique.

## Background

Traditional endovascular approaches pose significant challenges for ruptured aneurysms that are inaccessible by microcatheterization. These rare aneurysms usually exhibit a blister-like appearance with a narrow neck or tortuous parent artery. Recently, a few case reports and small case series have demonstrated that pure micro-guidewire electrocoagulation can serve as a practicable and effective option for aneurysms that cannot be catheterized with a microcatheter [1–3]. During electrocoagulation, the micro-guidewire tip acts as an anode, attracting negatively charged blood particles and promoting thrombosis within the aneurysm cavity. To date, the existing case studies have described satisfactory immediate and durable outcomes, with no observed instances of aneurysm recurrence after initial total occlusion [1, 4–6]. However, more research evidence is warranted to validate this promising new therapy.

In this report, we present a rare case of ruptured aneurysm of the A1 segment which was found to be recanalized 5 weeks after initial obliteration by endovascular electrocoagulation. Eventually, the recurrent aneurysm was successfully obliterated by surgical clipping.

## Case presentation

This report was prepared following the CAse REport (CARE) guidelines [7]. A 51-year-old female was admitted for acute-onset headache accompanied by nausea and vomiting. She had a history of hypertension for 3 years. The patient was neurologically intact except for nuchal rigidity (Hunt and Hess grade II).

**Fig. 1:**
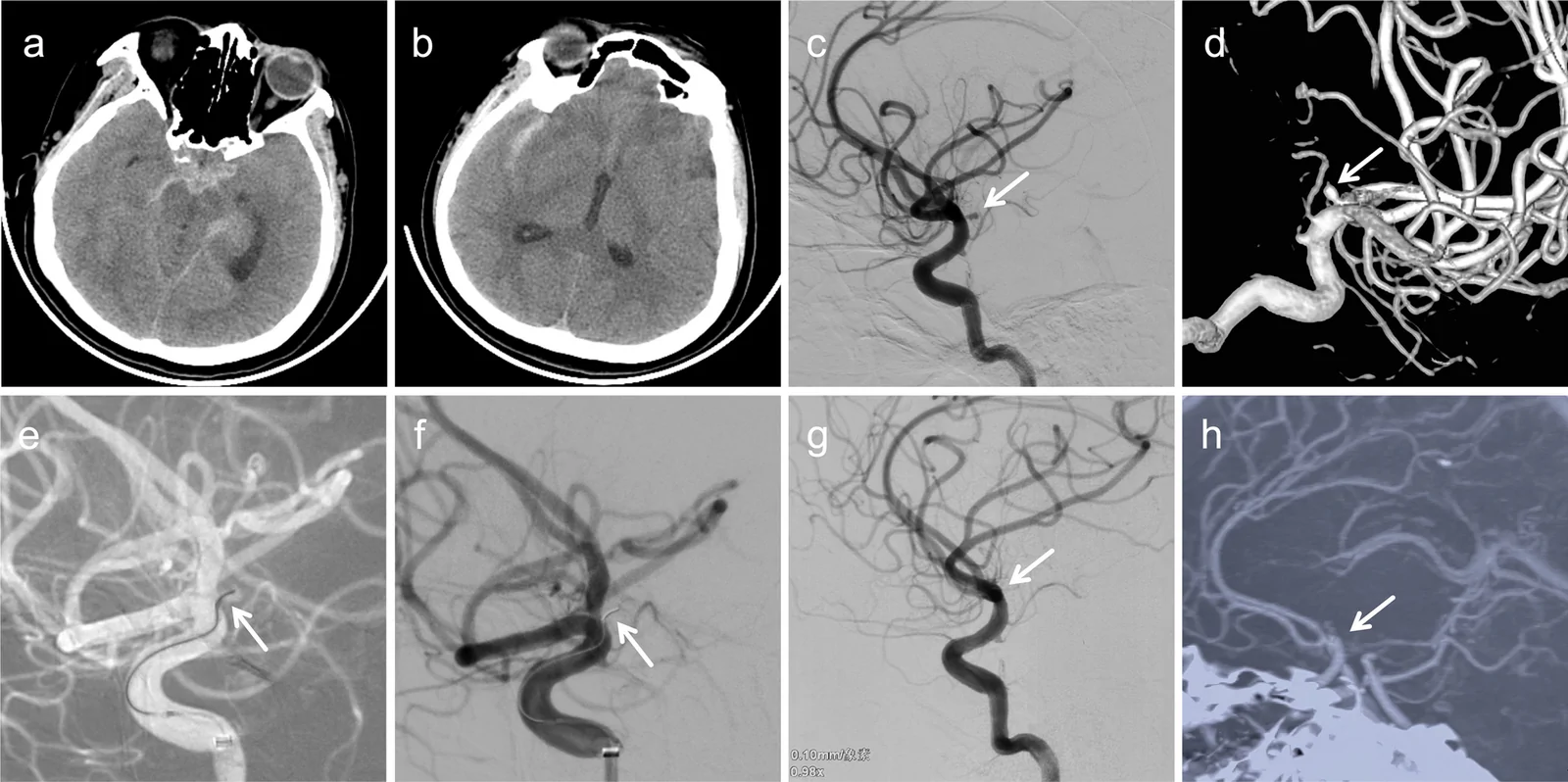
**a**, **b** Cranial CT showed the presence of a subarachnoid hemorrhage. **c**, **d** Lateral angiography and 3D-RA demonstrated a tiny aneurysm (arrow) arising from the A1 segment of the right anterior cerebral artery. The aneurysm exhibited a blister-like appearance with a narrow neck (arrow). **e** The tip of the Traxcess-14 guidewire was positioned within the aneurysm cavity, and the microcatheter was advanced close to the neck of the aneurysm (arrow). **f** After 5 cycles of electrocoagulation, the aneurysm opacification disappeared (arrow). **g** Lateral angiography repeated 20 min later demonstrated complete aneurysm occlusion. **h** Cranial CTA performed 2 days after procedure showed no opacification of the aneurysm

Emergency head computed tomography (CT) demonstrated diffuse subarachnoid hemorrhage, predominantly distributed in the interpeduncular cistern and the right sylvian fissure. Subsequent digital subtraction angiography (DSA) revealed a tiny aneurysm located at the origin of the right anterior cerebral artery (ACA). The aneurysm exhibited a blister-like appearance, measuring approximately 0.9 × 2.5 mm, having a narrow neck less than 0.5 mm (Fig. 1). This aneurysm was not of a common type and therefore did not undergo immediate treatment in the local center.

One day after the onset of SAH, the patient was referred to a tertiary center for management. Endovascular coil embolization was initially intended to be performed. Loading doses of aspirin (300 mg) and clopidogrel (300 mg) were administered 2 h prior to the procedure in case stent placement was required. During the procedure, an Echelon-10 microcatheter (ev3 Neurovascular, Irvine, USA) was used. Although the Traxcess-14 guidewire successfully reached the aneurysm, the microcatheter consistently failed to navigate into the aneurysm cavity due to the narrow neck and challenging angle. Thus, micro-guidewire electrocoagulation was selected as the salvage therapy. After the tip of the Traxcess-14 guidewire was positioned within the aneurysm cavity, the microcatheter was advanced to the vicinity of the aneurysm neck. Subsequently, the proximal end of the micro-guidewire was connected to the Solitaire stent detachment system (ev3). Energy delivery was set to 4.0 V and 1.0 mA current, with each electrocoagulation cycle duration for 1 min. After each electrocoagulation, angiography was repeated to assess the aneurysm opacification. After 5 cycles of electrocoagulation, the aneurysm opacification disappeared. An additional angiography was performed 20 min later to confirm the complete occlusion of the aneurysm. Postoperatively, the patient made a full neurological recovery without any new deficits. A follow-up computed tomography angiography (CTA) obtained 2 days after the procedure demonstrated no opacification of the aneurysm. The details of endovascular procedure are shown in Fig. 1. To ensure therapeutic efficacy, the patient was advised to undergo early surveillance angiography at 1 month after procedure.

**Fig. 2:**
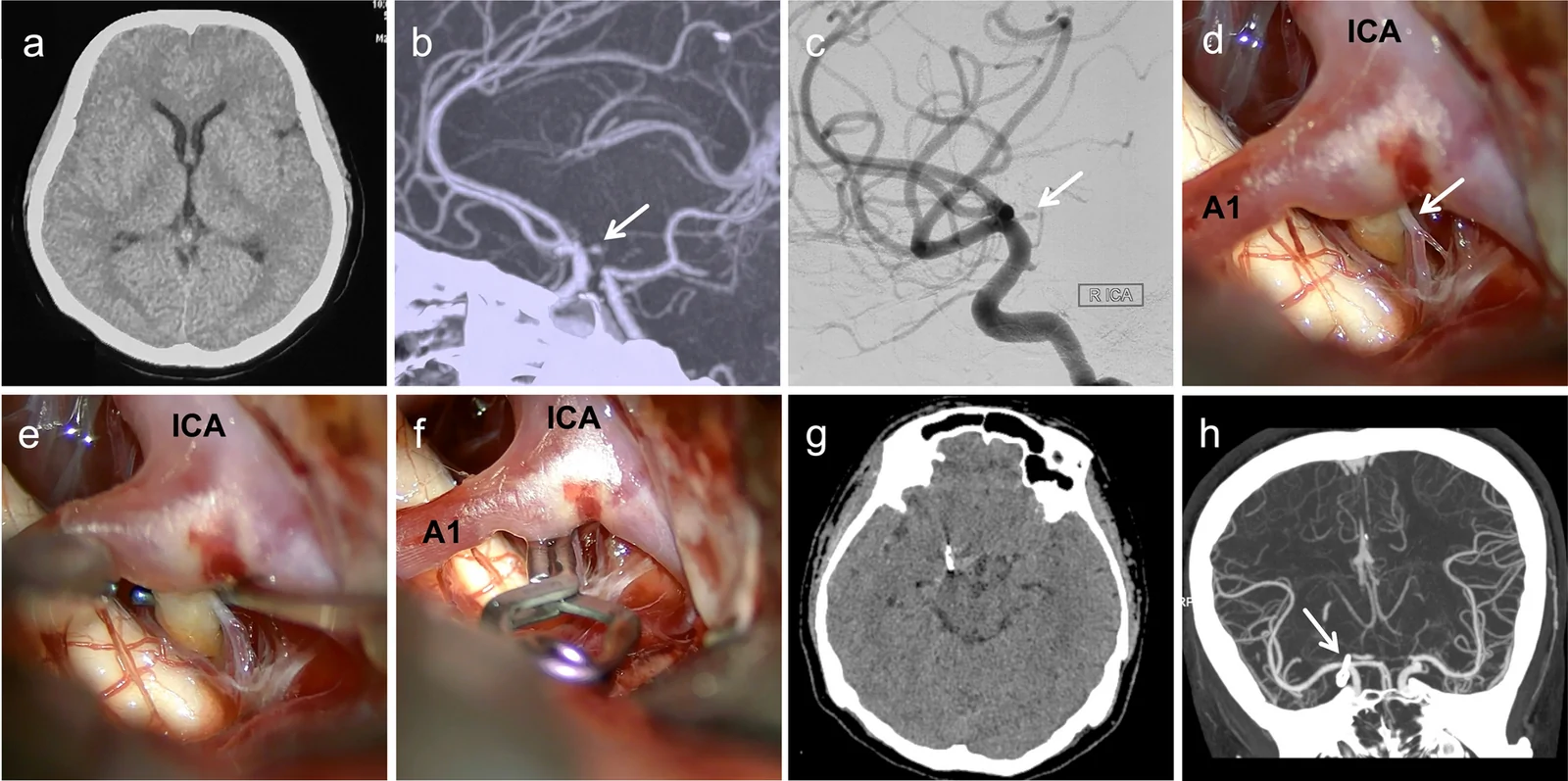
**a** Five weeks after procedure, a follow-up CT showed complete resolution of the subarachnoid hemorrhage and no evidence of ischemic foci. **b** However, the follow-up CTA indicated recanalization of the aneurysm at the original site (arrow). **c** Subsequent angiography confirmed the aneurysm recurrence (arrow). **d** The intraoperative microscopic view (via right frontal approach) during cranial surgery showed that the recurrent yellowish aneurysm originated from the dorsal wall of proximal A1. The aneurysm neck involved the origin of a perforator, of which the vessel lumen appeared thrombosed (arrow). **e**, **f** After neck dissection, the aneurysm was completely clipped. **g**, **h** One month after surgery, the follow-up CT showed no new lesions, and CTA demonstrated good positioning of the aneurysm clip (arrow)

However, a follow-up CTA at 5 weeks postoperatively showed recanalization of the aneurysm at the original site, which was subsequently confirmed by DSA (Fig. 2). The patient was referred to our center for retreatment. In order to achieve a more definitive therapeutic effect, we opted for surgical clipping instead of repeat endovascular therapy. A lateral frontal approach was employed. During the operation, after elevating the base of the frontal lobe to expose the carotid cistern, the recurrent yellowish aneurysm was found at the dorsal side of the proximal A1. We observed that the aneurysm neck derived from the junction of a small perforating branch and the parent artery, which was not revealed in prior angiogram. The lumen of the perforator appeared to have thrombosed. The detailed intraoperative microscopic view is presented in Fig. 2. After careful dissection of the neck, the aneurysm was completely obliterated with a straight mini clip. The patient made a favorable recovery exhibiting no neurological deficits and was discharged from the hospital 1 week after surgery. Follow-up CTA performed a month postoperatively demonstrated persistent obliteration of the aneurysm and good positioning of the clip (Fig. 2). The patient is scheduled to undergo a follow-up DSA at 6 months postoperatively.

## Discussion

Aneurysms at the A1 segment of ACA are considered rare lesions, representing approximately 1% of all intracranial aneurysms [8, 9]. Surgical clipping is often challenging for A1 aneurysms because of the anatomical variability and presence of important perforators in this location. Particularly for postero-superiorly directed aneurysms, the aneurysm sac is frequently buried in orbitofrontal cortex and enmeshed within surrounding perforators, thereby leading to increased risk of intraoperative aneurysm rupture and perforator injury [8]. By contrast, endovascular treatment for these aneurysms is also difficult and often hazardous [10]. The microcatheter positioning and support can be quite difficult, due to the predominantly posterior direction and the sharp turning angle of the microcatheter from the parent artery. Therefore, some advanced techniques, such as complex microcatheter shaping and microcatheter looping technique, frequently have to be used for accessing these aneurysms. In the present case, surgical clipping was not considered the first choice at the outset due to the following considerations. First, during the acute hemorrhagic phase, the difficulty of intraoperative retraction and exposure is significantly increased. Second, we believed that the blister-like appearance suggested a possible pseudoaneurysm, which heightened the risk of intraoperative rupture. However, during the endovascular procedure, multiple attempts at catheterization of the aneurysm sac were unsuccessful; thus, pure electrocoagulation was performed to obliterate the aneurysm. Fortunately, the patient made a favorable recovery without rebleeding.

It has been reported that endovascular electrocoagulation can be a promising alternative for certain ruptured aneurysms that are refractory to microcatheter access [1–3]. The pathophysiological mechanism of electrocoagulation is hypothesized to involve promoting thrombus formation and triggering vascular wall endothelial remodeling [6]. Before electrocoagulation, the micro-guidewire tip was navigated into the aneurysm cavity, and the microcatheter was advanced to the immediate vicinity of the aneurysm neck to confine the effect within the aneurysm lumen. Previously, Lu et al. described the largest case series of seven microcatheter-inaccessible ruptured aneurysms, which were successfully occluded by pure micro-guidewire electrocoagulation without any rebleeding or aneurysm recurrence during the follow-up. [1] Additionally, several recent case reports have also demonstrated the efficacy and safety of this technique for treating ruptured perforator aneurysms where conventional therapeutic approaches have failed [5, 6, 11, 12]. To date, in the limited published series totaling 19 patients, no recurrence has been reported [1, 5, 6, 11, 12]. Our report is the first documented recurrence after endovascular electrocoagulation, which raises the concern about the durability of this endovascular technique and highlights the importance of early angiographic follow-up after electrocoagulation. It is considered that flow diversion (FD) represents another therapeutic option for this recurrent aneurysm [13]. However, in one case, the FD stent can be released from ACA to ICA, but the covered MCA origin will delay the filling of the MCA territory, risking serious ischemic stroke due to hypoperfusion [14]. In another case, the FD stent is placed from MCA to ICA leaving the aneurysm neck uncovered, but there is still a risk of rebleeding after procedure due to the uncertain flow diverting efficacy of this approach [15]. Consequently, open surgery was selected to completely eliminate the risk of rebleeding.

Surgical clipping was proved to be a successful treatment for this case, and it provided a direct view of the pathological changes following electrocoagulation therapy. The aneurysm was seen arising from the origin of a small perforating branch. It was observed that the aneurysm wall had developed fibrous hyperplasia and become opaque, which could be related to the inflammatory response caused by thermal effect. We also found that the origin of perforating artery adhered to the aneurysm had become occluded, which raised another concern about the risk of perforator infarction associated with electrocoagulation. Past studies have reported the incidence of acute lacunar infarctions related to electrocoagulation procedure [1, 6]. Therefore, when selecting this technique, the risks of damaging neighboring perforating vessels must be considered.

We hypothesize that some possible factors contributed to the clot lysis and aneurysm recurrence. One factor is that the persistent hemodynamic stress on the dorsal aspect of the A1 segment could disrupt the unstable fresh thrombi. Additionally, while initial occlusion was achieved, the energy of electrocoagulation might be insufficient to induce durable endothelial fibrosis and remodeling. The parameters (4.0 V/1.0 mA) were selected based on reported protocols in the literature, which have demonstrated safety and efficacy [1, 2, 6]. However, in the present case, while angiography showed no excessive thrombosis or compromised blood flow within the parent artery, the direct view revealed the occlusion of an adjacent perforator, likely related to thermal spread. Therefore, further research is warranted to determine the optimal electrocoagulation parameters to balance therapeutic effect and collateral damage.

There were some limitations in our report. First, the exact timing of aneurysm recanalization was not known, due to the absence of intermediate imaging between postoperative day 2 and week 5. Second, while neurologically intact, small lacunar infarcts related to perforator occlusion could not be fully excluded, given the absence of postprocedural diffusion-weighted imaging. Third, the precise mechanism underlying aneurysm recurrence after electrocoagulation is unclear and remains to be studied.

## Conclusion

Despite achieving immediate angiographic occlusion, aneurysms treated by endovascular electrocoagulation carry a risk of recanalization. This underscores the necessity of close angiographic follow-up to ensure the efficacy of this technique.

## Data Availability

No datasets were generated or analysed during the current study.

## Abbreviations

CARE: CAse REport
CT: Computed tomography
DSA: Digital subtraction angiography
ACA: Anterior cerebral artery
CTA: Computed tomography angiography
FD: Flow diversion
ICA: Internal Carotid Artery
MCA: Middle Cerebral Artery
